# An Epigenetics-Based Hypothesis of Autoantigen Development in Systemic Lupus Erythematosus

**DOI:** 10.3390/epigenomes4020006

**Published:** 2020-04-23

**Authors:** Wesley Brooks

**Affiliations:** Department of Chemistry, University of South Florida, Tampa, FL 33620, USA; wesleybrooks@usf.edu; Tel.: +1-813-758-5059

**Keywords:** epigenetics, lupus, polyamines, X chromosome, nucleolus, autoantigens, autoimmune disease, cGAS, STING, Alu

## Abstract

Currently, we have a limited understanding of mechanisms leading to systemic lupus erythematosus, but we know that genetics, environmental factors, and epigenetics contribute to the disease. One common aspect of the various environmental triggers is that they can cause cellular stress. When extraordinary stress occurs, such as viral activation, a cell’s response can include increased nucleolar volume and activity to produce more machinery (e.g., ribosomes) to help the cell recover. However, nucleolar expansion can disrupt the epigenetic control in neighboring heterochromatin that comprises the nucleolar shell. This disruption can open underlying vulnerabilities that provoke an autoimmune reaction. Here, we review the “X chromosome-nucleolus nexus” hypothesis, which explains how nucleolar stress can disrupt epigenetically silenced chromatin, especially the neighboring inactive X chromosome (aka the nucleolar satellite). Chromatin disruption can lead to the expression of sequestered DNA, such as *Alu* elements and fully functional *LINE-1* reverse transcriptase genes. In addition, *Alu* transcripts can disrupt the nucleolar structural integrity, leading to nucleolar disintegration. Such disintegration can leave nucleolar components and products in autoantigenic forms, such as abnormal conformations or incomplete macromolecular assemblies. Recent research on DNA sensing pathways can now be incorporated into the hypothesis to provide further details explaining how autoantibodies to endogenous nucleic acids arise.

## 1. Introduction—The Complexity of Autoimmune Diseases

Autoimmune diseases, in which the immune system attacks one’s own body, are some of the most complex diseases confronting modern medical research since autoimmune diseases are multifactorial, presenting a broad array of triggers, symptoms, and autoantigens. Environmental factors, genetics, and epigenetics all contribute to autoimmune diseases. Autoimmune diseases vary widely with regards to the onset and progression of symptoms making each autoimmune disease patient somewhat unique even though an estimated 5–10% of the population suffers from one or more autoimmune diseases. The American Autoimmune Related Diseases Association website lists 151 such diseases [1] and there may be another 40 diseases that are suspected of autoimmune involvement either underlying the disease (e.g., possibly Alzheimer’s from aging of the blood-brain barrier [2]) or as a secondary event (e.g., occasional autoimmune reactions arising with some cancers or cancer therapy [3,4]). However, there is an overlap between many of the autoimmune diseases such as Sjögren’s syndrome (SjS), which can be a primary disease or it can be secondary to rheumatoid arthritis (RA) [5], systemic lupus erythematosus (SLE) [6], or multiple sclerosis (MS) [7]. In fact, co-occurrence of the diseases has been reported in one study to be approximately 34% [8]. This suggests that there may be shared mechanistic aspects among some of these diseases.

The complexity of autoimmune diseases is also apparent from the diversity of autoantigen types that can occur. SLE, which exhibits the greatest assortment of autoantigens among the autoimmune diseases, now has over 180 autoantigen types as potential targets [9]. However, the specific autoantigens targeted in each SLE patient can vary suggesting subsets of related autoantigens (e.g., ANA; antinuclear autoantibodies) and nuances in mechanisms. Other autoimmune diseases can also target some of these same SLE-associated autoantigens. For example, both SLE and RA can have autoantibodies targeting Z-DNA, a normally transient conformation of DNA with left-handed coiling of the DNA double helix as opposed to the predominant form, right-handed coiling B-DNA [10]. Another example is the occurrence of anti-Ro and anti-La autoantibodies in both SLE and SjS [11,12].

Some of the complexity of autoimmune diseases hindering our understanding arises from our efforts to define the diseases, in effect, classification criteria. Although autoimmune diseases have been recognized since before the 1960s, refinements to the classification criteria have been through several iterations. Diagnostic criteria for SLE, for example, were first described in 1971 but major revisions have occurred in 1982, 1997, and again in 2012 [13]. Currently another revision is under development to incorporate new insights on the earlier events in the SLE onset [14]. The slow appearance and variability of symptoms can delay a definitive diagnosis, which makes it difficult to assemble cohorts for clinical trials and for thoroughly defining characteristics of a specific autoimmune disease.

For this discussion, we will focus on SLE, which is considered to be the prototypical autoimmune disease since SLE presents much of the breadth of symptoms and autoantigens seen in autoimmune diseases. Genetics has a contributing role in the SLE and some specific genes have been identified that have a role in some SLE cases but, for this discussion, we will focus on epigenetics. What follows is a presentation of the “X chromosome-nucleolus nexus” hypothesis, which can be considered as an antigen-driven hypothesis, i.e., the immune system is reacting to the appearance of abnormal material [15,16,17]. The abnormal material could be: Endogenous material in abnormal conformations (e.g., cruciforms); abnormal or missing modifications (e.g., hypomethylated DNA); exposure of normally sequestered epitopes; macromolecular assemblies of endogenous material misassembled and/or out of normal context; pathogenic exogenous material; or exogenous material that mimics endogenous material. Persistence of the autoantigenic material and epitope spreading can then lead to an autoimmune reaction to the more abundant endogenous material in its normal context. The “X chromosome-nucleolus nexus” hypothesis explains how abnormal material can arise as autoantigens in cells upon disruption of epigenetic control during cellular stress. Some new ideas are discussed that expand the hypothesis with regards to DNA sensing and autoantigenic nucleic acids. 

## 2. Components of the “X Chromosome-Nucleolus Nexus” Hypothesis

### 2.1. The Hypothesis in Brief

The basic concept of the hypothesis is that an extensive nucleolar stress response to an environmental insult (e.g., EBV infection [18] or lupus-inducing drugs such as hydralazine [19]) or an accumulation of damage from multiple episodes of stress response can lead to disruption of heterochromatin neighboring the nucleolus [16]. The nucleolus does not have its own membrane but is delimited by a shell of heterochromatin. Heterochromatin contains suppressed genes that are tightly packaged to keep them silenced but, with disruption of the heterochromatin when a nucleolus expands in a stress response, some previously suppressed gene alleles in the heterochromatin could be opened and potentiated for activation. In a nucleolar stress response, the nucleolus typically expands rapidly by restructuring its heterochromatic shell in order to increase its capacity to create more transfer RNAs (tRNAs) and ribonucleoproteins (RNPs; e.g., ribosomes and spliceosomes), which will be needed for the cell to recover from the stress. If a virus is involved in the stress, either as a trigger of the event or becoming active due to the event initiated by some other trigger, it could be an advantage for the virus if the host cell’s nucleolar capacity increases since the virus would benefit from more ribosomes and tRNAs to translate viral proteins. Particularly vulnerable to disruption during nucleolar stress is the heterochromatic inactive X chromosome (Xi), which is frequently associated with the nucleolus as part of the nucleolar shell [20]. In fact, the Xi was originally termed the “nucleolar satellite” before it was determined to be a chromosome. It was given the term sex chromatin (aka the Barr body) when it was found to be a regular feature in female cells but not in male cells [21]. Subsequently, the Barr body was recognized as being an X chromosome [22]. Soon after, the concept of the inactive X chromosome, Xi, was proposed as an explanation for the structure [23]. A possible involvement of the Xi could help explain the female predominance among SLE patients. 

Disruption of chromatin can release negative supercoiling stress stored in nucleosomes (each nucleosome stores approximately one negative supercoil). The released negative supercoiling stress can flux through the DNA and potentially be stabilized in alternate DNA forms such as Z-DNA or cruciforms, which are both considered to be alternate forms of negative supercoiling stress storage. These usually transient conformations, if stabilized, could persist long enough and in sufficient quantity to be interpreted by the immune system as autoantigenic [24]. Keep in mind that *Alu* elements have potential for cruciform formation and there are more than 10^6^ *Alu* elements in the human genome. In addition, chromatin disruption could open previously sequestered gene alleles for expression that can lead to the loss of control of gene product levels. As a result, there can be overexpression of some genes and increased or altered activity of the resulting RNAs and proteins. An especially vulnerable section of chromatin is the short arm of the Xi (Xp). Since establishment of X chromosome inactivation reaches the Xp last and roughly 35% of Xp genes avoid inactivation, this would place much of the Xp at the surface of the dense heterochromatic Xi. The Xi is the last chromosome to be repackaged in the S phase (sometimes not even completed until the early G2 phase), which puts the Xi and especially the Xp at risk of incomplete repackaging before mitosis occurs. Due to this late completion of replication and heavy demands for methylation, the Xi is particularly vulnerable to depleted levels of S-adenosylmethionine (SAM) needed for methylation and it is vulnerable to complications that must be resolved in fragile sites, such as alternate DNA conformations and DNA damage. Without resolution, there could be chromatin fragmentation and/or escape of genes from X inactivation. Contributing to this, scenarios such as Z-DNA and cruciforms can present sites of DNA strand separation (such as in the change in DNA base stacking in B-Z junctions) that are vulnerable to breakage, mutations and insertions, or they can simply hinder rates of DNA repair, transcription, or replication. 

The Xi, being tightly condensed heterochromatin, has high levels of negative supercoiling stress stored in its nucleosomes that could be released during disruption. Moreover, Xp has a high concentration of CpG-rich *Alu* elements which, if transcribed by the abundance of RNA polymerase III (RNA pol III) typically near the nucleolus, could disrupt the nucleolus’s integrity and disrupt the production and assembly of macromolecular complexes occurring in the nucleolus [17,25]. Such disruption causing nucleolar dysfunction can even lead to fragmentation of nucleoli. Nucleolar disruption could leave proteins and RNAs in abnormal conformations and ribonucleoproteins could be in misassembled or incomplete complexes. Each of these abnormal items could provoke an autoimmune reaction. Many of the autoantigens targeted in autoimmune diseases are, in fact, components or products of the nucleolus, at least transiently (Table 1). These may be abnormal macromolecular assemblies, abnormal conformations, or cryptic epitopes (i.e., normally hidden in the structure) in abundance for which immunological tolerance does not exist. Epitope spreading can shift the autoimmune response from the initial autoantigenic epitopes to include neighboring normal epitopes that are in much greater abundance on normal proteins or RNA throughout the body. Other consequences can occur originating from disruption of the Xi and nucleolus. These will be discussed later.

### 2.2. The Nucleolus: Functions and Stress

Nucleolar functions and structure have been reviewed recently [29,30,31,32]. Among the many nucleolar functions, nucleoli are involved in DNA repair, chromatin replication, centromere assembly, and assembly of ribonucleoprotein complexes including ribosomal subunits, spliceosomal subunits, tRNAs, and signal recognition particles (SRP) [33,34]. Those nucleolar functions that deal with DNA (repair and replication) would be strictly monitored and controlled in the cell by check points, ATP levels, and apoptotic triggers but those functions that deal with ribonucleoprotein assembly can actually increase in response to cellular stress to create the machinery needed to facilitate cellular recovery. The response can also include remodeling of the heterochromatic shell that delimits the nucleolus from the rest of the nucleus. Such remodeling can expand the nucleolar volume to accommodate increased nucleolar activity.

With regards to the nucleolar structure, when a cell is stained with DAPI to highlight the cell’s DNA, the nucleolus appears as a large sparse area in the cell’s nucleus since the nucleolus has little DNA in it. However, the nucleolus does contain some DNA, primarily the ribosomal genes in nucleolar organizing regions (NORs), found in chromosomes 13, 14, 15, 21, and 22, and the nucleolus is associated with chromosomes when centromeres are assembled [35]. After assembling centromeres, the nucleoli disappear during mitosis as the cell divides, but the nucleoli reappear in the daughter cells by forming around NORs. The nucleolus is also purported to have roles in maintaining X chromosome inactivation (XCI) and in DNA repair [20]. For the most part, however, the nucleolus’s most defining function is folding of RNAs and assembling ribonucleoprotein complexes, so the nucleolus contains primarily proteins and RNA. We can consider a nascent RNA molecule as primarily a long linear entity as it is transcribed, even though the RNA may be transiently coiled and associated with chaperone proteins, such as the SSA/Ro and SSB/La proteins. In the nucleolus, the RNA will go through multiple folding steps and binding of proteins to yield a functional ribonucleoprotein complex. Thus, the RNA needs the large open volume of the nucleolus for the RNA’s transition from a randomly coiled entity to a properly folded three-dimensional complex. This need for space explains the sparsity of DNA and chromatin in the nucleolus as it is assembling ribonucleoproteins. However, the nucleolus does not have a defined membrane separating it from the rest of the nucleus but rather the nucleolus is a cavity surrounded by a heterochromatic shell, which can include the Xi [36,37]. Proteins in the nucleolus, such as nucleolin and nucleophosmin, associate with structural RNAs, nuclear lamins, and the surrounding heterochromatin to establish the shell and to maintain the nucleolar integrity as a ribonucleoprotein assembly site, which includes not just folding of the RNA transcripts but can also include refolding as needed to provide quality control. The nucleolar interactions and structural components are currently under intense study to gain a detailed understanding of the processes occurring in the nucleoli. 

Most of the RNA transcripts that are assembled into cellular machinery in the nucleolus are transcribed by RNA pol I and RNA pol III as opposed to RNA pol II, which creates protein-coding pre-messenger and messenger RNAs (mRNAs). In fact, RNA pol I, which transcribes large ribosomal RNAs (rRNA), and RNA pol III, which transcribes tRNAs, 5S rRNA, the core signal recognition particle 7SL RNA, and the spliceosomal U6 snRNA, together account for approximately 60% of cellular transcription [38]. Thus, the nucleolus typically has an abundance of RNA pol III enzyme in close proximity, especially when the nucleolus is very active. RNA pol I and RNA pol III do not require ATP for transcription whereas RNA pol II requires ATP for DNA helicase to separate the double stranded DNA (dsDNA) strands. Therefore, if ATP levels drop during cellular stress, the RNA pol II activity would decrease but the RNA pol I and RNA pol III activity can continue. 

**Figure 1:**
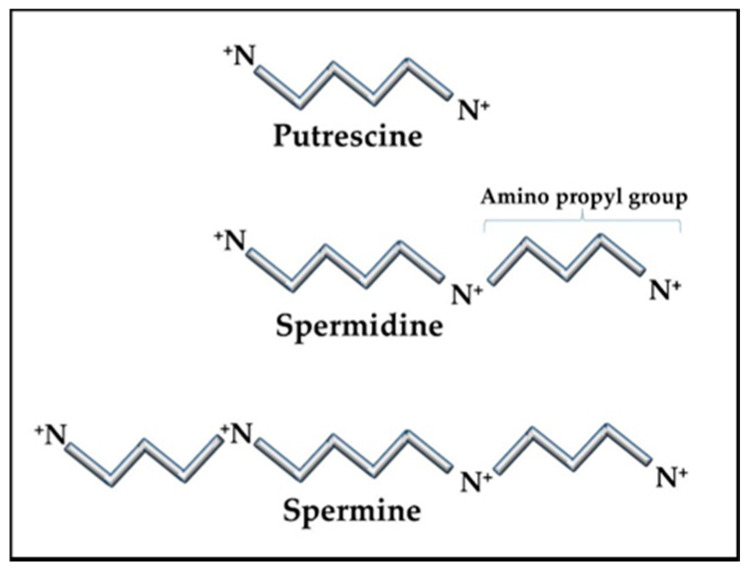
Polyamine structures. The polyamine precursor diamine, putrescine, is usually kept at only trace amounts in cells as a means of controlling overall polyamine levels. Amino propyl groups from decarboxylated S-adenosylmethionine (SAM) are added to putrescine to create spermidine and spermine. Spermidine and spermine concentrations can vary in cells but are typically in the sub-millimolar to millimolar range, reaching the higher levels during stress and replication.

The RNAs and RNPs processed in the nucleolus require extensive folding and timely ordered binding of protein components as the accommodating RNA topology appears. The result of these stepwise processes is the formation of proper and complete RNP complexes. For example, the SRP has a core RNA transcript, 7SL RNA, which is folded and then binds six proteins [39]. RNA folding is a particularly difficult process since the RNA is highly anionic and, therefore, has limited flexibility due to self-repulsion. To overcome this difficulty, the nucleolus contains high levels of the cationic polyamines, spermidine, and spermine (+3 and +4 in vivo, respectively), to act as counter ions in the folding process (Figure 1). The polyamines have many essential functions in the cell. In the nucleolus, the polyamines are essential to assist in folding the RNA transcripts through many transient interactions between the polyamines and RNA with the polyamines masking the RNA’s anionic charges sufficiently for the RNA to fold. This allows the RNA to form stable intra-strand interactions and to present the proper RNA topology for protein binding. Improper or incomplete conformations could generate RNPs that are not functional and that could provoke an autoimmune reaction. Specific proteins (e.g., SSA/Ro) are also involved in chaperoning the RNA pol III transcripts and assisting in folding and refolding as needed.

The nucleolus is very dynamic in its size, function, and contents. More than 4500 proteins are associated with the nucleolus, but the nucleolar proteome components can rapidly change as priorities change and proteins are exchanged back and forth with the cytoplasm [40,41]. There can be multiple nucleoli in a cell, and they can take up as much as 25% of the volume in the nucleus. The dynamic changes in nucleolar size are directly related to the polyamine levels [42,43]. The nucleolus typically expands as polyamine levels increase since increased RNA folding and RNP assembly require more polyamines and more volume in which to proceed. In the case of viral induced cellular stress, an active virus will typically induce increased polyamine synthesis as an initial step in order to increase the nucleolar capacity to handle the viral demands. One example is EBV, which induces increased c-MYC activity that increases transcription of polyamine enzymes ornithine decarboxylase (ODC), spermidine synthase (SRM), and spermine synthase (SMS), and thereby increases polyamines [44,45]. The polyamines then induce increased nucleolar size and activity [43]. Other viruses or environmental factors can, concurrently or subsequently, add to the burden placed on nucleoli to respond to stress.

### 2.3. Polyamines

**Figure 2:**
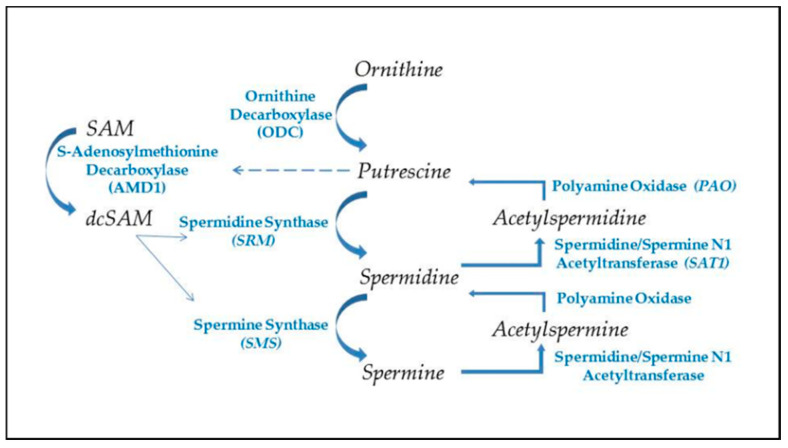
Polyamine synthesis and recycling. Polyamine synthesis begins with decarboxylation of ornithine from the urea cycle. This generates putrescine, which allosterically induces increased conversion of S-adenosylmethionine to decarboxylated SAM (dcSAM), which will provide an amino propyl group for synthesis of spermidine and spermine. Recycling of polyamines occurs by acetylation of spermine or spermidine, using acetyl CoA, followed by oxidation to generate spermidine or putrescine, respectively. Production of putrescine by recycling provides an alternate route for initiating further polyamine synthesis.

The ubiquitous polyamines, spermidine, and spermine, and their precursor, putrescine (Figure 1) are tightly controlled in cells due to their importance in many cellular functions and their competition with cellular methylation for S-adenosylmethionine (SAM), the cell’s methyl donor [46,47]. Polyamines also compete with the synthesis of some amino acids since putrescine is generated from ornithine, which originates from arginine in the urea cycle. Cells have means of interconversion of arginine, proline, and ornithine so increased polyamine synthesis could aberrantly affect this balance [48]. In addition to assisting in RNA folding and RNP assembly in the nucleolus, polyamines are important in transcription, translation, replication, control of ion channels, cell adhesion, and signal transmission (e.g., neuron synapses). Therefore, polyamine levels must be tightly controlled to avoid over activity of processes, such as replication in hyperplasia, and to avoid unwanted decreases in SAM that could affect epigenetic gene silencing and intracellular translocation of proteins and nucleic acids. Whereas spermidine and spermine levels are kept around 1 mM or less, putrescine is usually kept at trace levels since it can allosterically increase decarboxylation of SAM by the enzyme SAM decarboxylase (AMD1) as a preliminary step in polyamine synthesis (Figure 2) [49]. The decarboxylated SAM cannot be used for cellular methylation. Abnormally increased polyamine synthesis drawing on SAM levels could have a role in the hypomethylation and altered gene expression seen in some autoimmune diseases and cancers. 

The unique characteristics of polyamines make them invaluable in intracellular functions, especially in manipulation and control of nucleic acid conformations. The polyamines have high cationic states in vivo (putrescine +2; spermidine +3; spermine +4) but the charges are spread out over their length (putrescine ~ 8 Å; spermidine ~12 Å; spermine ~16 Å) and, since they do not have any double bonds, they are quite flexible. These features give them the unique dynamics needed to serve as counter ions to reduce the self-repulsion in negatively charged nucleic acids to assist in folding and conformation stabilization of nucleic acids. Polyamines are also essential in DNA compaction in chromatin [50]. One advantageous aspect of the polyamine structures is that the positively charged nitrogens are separated by carbons such that water molecules cannot organize in multiple levels, so the polyamine’s nitrogens can be positioned closer to negatively charged phosphates in nucleic acids. Additionally, the nitrogens maintain their relative positioning along the length of the polyamine/nucleic acid complex due to their covalent bonds within the polyamine. On the other hand, a cation such as Mg^+2^ has more charge as a single point but it is usually shielded by layers of water molecules that have organized around it making the Mg^+2^/water a bulkier counter ion and the Mg^+2^ has no covalent connection with other cations associated along the nucleic acid. The interactions of polyamines with nucleic acids are usually multiple but transient during the stepwise folding of RNA transcripts and binding of any proteins in nucleoprotein complexes [51]. However, in some situations, they can remain as a component of the final product, such as in tRNAs [52]. In addition, polyamines can modulate conformations of higher order chromatin [53] and stabilize Z-DNA [54]. Polyamines are also very effective in facilitating the transition of B-DNA to cruciforms [55].

Since the nucleolus is a major site of RNA folding and chromatin remodeling, polyamines are important in nucleolar functions and dynamics. In fact, changes in nucleolar size and functioning are directly related to changes in cellular polyamine levels and the highest concentrations of polyamines in the cell are found in the nucleolus [43,56,57]. Most polyamines are noncovalently bound to nucleic acids and phospholipids but sudden changes in free polyamine levels can impact many functions throughout the cell.

Polyamine synthesis (Figure 2) begins with the conversion of ornithine to putrescine by the enzyme ornithine decarboxylase (ODC), one of the most tightly controlled enzymes in the cell with a rapid turnover of the ODC transcript and protein, and an antizyme that suppresses the ODC activity [58]. Polyamine synthesis is further regulated by putrescine’s control of AMD1′s post-translational processing and allosteric activation [59]. As ODC generates putrescine, the putrescine increases AMD1 conversion of SAM to decarboxylated SAM (dcSAM), which provides an amino propyl group used in the conversion of putrescine to spermidine by the spermidine synthase. Another amino propyl group is used in the conversion of spermidine to spermine by the enzyme spermine synthase. Since an activated virus in a cell needs to have the viral RNA folded and viral proteins synthesized by the cell’s ribosomes, the virus will target the cell’s c-MYC to increase expression of polyamine synthesis genes [60]. The resulting increase in polyamines induces more nucleolar generation of ribosomes and tRNAs and the polyamines can assist in packaging of viral particles. Polyamine recycling provides an alternate means of controlling polyamine levels. Acetylation of polyamines is a first step in the export of cellular polyamines to reduce intracellular polyamine levels when needed. Acetylation of polyamines by spermidine/spermine N1 acetyltransferase (SAT1) followed by oxidation by polyamine oxidase (PAO) can reduce the levels of spermine and/or spermidine as needed. This has the effect of reducing the cationic character of the overall intracellular polyamine pool since the +4 spermine becomes +3 acetylspermine, which can be oxidized to +3 spermidine and the spermidine can become +2 acetylspermidine, which can be oxidized to +2 putrescine. Acetylation of spermidine and/or spermine could hinder the nucleolar stress response by lowering the cationic values of nucleolar polyamines and thereby hampering nucleolar RNA folding, tRNA creation, and reorganization of the heterochromatic shell to expand the nucleolus [61]. Creation of putrescine via recycling can induce more polyamine synthesis due to the allosteric effect of putrescine on AMD1. This may benefit the cell by reducing some of the draw on ornithine levels, but it can initiate a wasteful use of SAM by continual cycling through polyamine synthesis and recycling and thereby needlessly reducing acetyl-CoA needed for protein acetylation and reducing SAM needed for DNA methylation [62]. In addition, acrolein, a cytotoxin, can be generated by oxidation of spermine, spermidine, or putrescine [63,64]. Moreover, in the case of SAT1, it can undergo super induction with an increase in activity of several hundred-fold in response to reactive oxygen or nitrogen species (ROS, RNS). As SAT1 reduces the spermine and spermidine levels, there is a risk of mitochondrial-induced apoptosis [65]. This abnormal polyamine recycling activity could be an especially critical problem for a cell that has had reactivation of SAT1 located at Xp22.1 on the short arm (Xp) of the Xi as proposed in the “X chromosome-nucleolus nexus” hypothesis. Normally SAT1 is inactive on the Xi but disruption of the Xi, such as from abnormal nucleolar expansion, could lead to expression, even super induction of SAT1 from both X chromosomes. Initially there could be an increase in polyamines from the triggering stress that increases the nucleolar size and activity but then SAT1 would reduce polyamine levels and types so that future nucleolar function and stress response are muted due to lower spermidine and spermine levels and possibly increased levels of acetylated polyamines with reduced cationic charges. This could lead to an increase in abnormally folded RNA and abnormal RNP assembly. Induction of SAT1 can also provide an alternate route that creates putrescine to feed into polyamine synthesis to create even more spermidine and spermine, which would interfere with the overall epigenetic control of chromatin due to the competition for acetyl-CoA and SAM. 

A contributing role for polyamines in autoimmune diseases is suggested by a variety of observations. First, elevated levels of polyamines have been observed in the urine of RA patients [66] and increases in SAT1, ODC, and putrescine have been reported for synovial fibroblasts in RA [67]. This has led to the proposal that SAT1 could be a new therapeutic target to exploit in RA [68]. With regards to ODC, diflouromethylornithine (DFMO; aka elfornithine) is an irreversible inhibitor of ODC. Treatment with 1% DFMO in water given to NZB/W mice (a lupus-prone model) reduced IgG and IgA levels in the mice and reduced spermidine and putrescine levels suggesting the inhibition of ODC as a promising avenue for SLE therapeutics [69]. As for SLE in humans, Kim et al. reported differences in plasma polyamine levels of SLE patients compared to controls with SLE patients showing significant decreases in spermine, spermidine, acetylspermidine, and acetylcadaverine while there was an increase in cadaverine [70]. Thomas et al. reported greater binding of SLE anti-DNA antibodies in the presence of spermine, suggesting a possible role of spermine in the autoantigen creation [71]. Another observation in SjS shows a close correlation of the intensity of the SjS episode with the appearance of acrolein conjugated proteins [72,73]. The acrolein could be a product of polyamine degradation or oxidation of polyamines that have been conjugated to proteins by the transglutaminase activity.

To some extent, the data on polyamines in autoimmune diseases seem contradictory. Increases in the main polyamines (spermidine and spermine) appear to be important since they relate to increased nucleolar capacity, chromatin condensation, and stabilization of alternate nucleic acid conformations. However, recycling of polyamines via acetylation and oxidation is also relevant since this alters the net counter-ion potential in the nucleolus and can lead to wasteful cycling through polyamine synthesis and recycling. Therefore, we should think of polyamine involvement in autoimmune disease mechanisms as being multifaceted and can initially cause an exaggerated nucleolar response when polyamine synthesis is induced but subsequent nucleolar responses and functioning may be muted and abnormal due to increased polyamine recycling. Additionally, the wasteful use of SAM can affect the DNA methylation needed for proper epigenetic control, such as maintaining silencing in the Xi. Moreover, the polyamines can stabilize potential autoantigens, such as Z-DNA.

### 2.4. The Inactive X Chromosome

**Figure 3:**
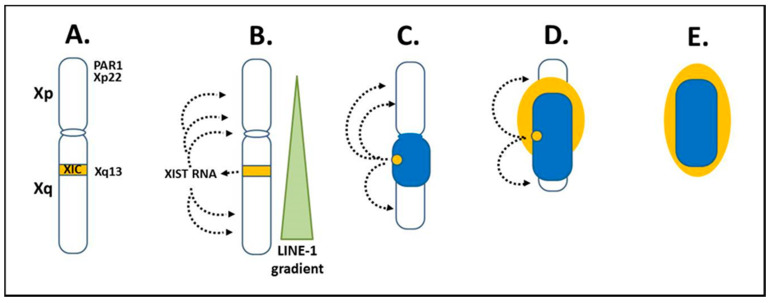
X chromosome inactivation. (**A**) X chromosome inactivation (XCI) initiates from the X inactivation center (XIC) at Xq13 of the X long arm (Xq). (**B**) X inactivation specific transcript (XIST) RNA is expressed from Xq13 on the X chromosome that will become the Xi. XIST RNA remains in the nucleus and binds along the future inactive X (Xi) anchoring in *LINE-1* elements. *LINE-1* elements are in a decreasing gradient from the Xq into the short arm (Xp), which coincides with more genes avoiding inactivation in Xp as *LINE-1* decreases. (**C**) The Xi begins to form as a dense heterochromatic body (dark blue) with primarily Xq genes at the core and later, as XCI moves to Xp, some Xp genes are inactivated. (**D**,**E**) Genes escaping inactivation (light yellow), primarily Xp genes, along with neighboring inactivated genes are at the Xi surface. Xp genes in the pseudo-autosomal region (PAR1) and Xp22 are particularly important in the “X chromosome-nucleolus nexus” hypothesis.

The “X chromosome-nucleolus nexus” hypothesis, first presented in 2015, proposed a role for the Xi in autoimmune diseases, such as SLE [15,16,17]. This was based on the increased risk for developing an autoimmune disease when there is more than one X chromosome in the cell, thus providing an explanation for the female bias in autoimmune diseases. For example, women (46, XX; 47, XXX; etc.) comprise 90% of SLE patients and the SLE risk is 14x greater in Klinefelter’s males (47, XXY) compared to normal males (46, XY) [74]. Since autoimmune diseases typically appear in adulthood, it suggests a cumulative and combined effect of X-linked epigenetic changes, genetic predisposition, and environmental stresses. This could include an accumulating loss of the epigenetic control of genes and elements on the Xi. The Xi is, in fact, a major epigenetic structure in the cell and the X chromosome inactivation (XCI) process is a major epigenetic event in female cells [75,76,77,78]. Since sexual differentiation is driven by the SRY gene on the Y chromosome and since most X-linked genes are not involved in sexual development, the female cells, with regards to most X-linked genes, need only one active X chromosome (Xa), such as in male cells, so that there is an equivalent X-linked gene expression in male and female cells. This is referred to as X-linked dosage compensation. XCI begins early in embryogenesis when there are less than one hundred cells. Each female cell will randomly inactivate one of its X chromosomes, either the paternally-derived X or the maternally-derived X. After that, each daughter cell will inherit the same inactivation pattern so that the adult will have areas where all cells have the paternally-derived X inactive and other areas where all cells have the maternally-derived X inactive. Once inactivated, the Xi appears as a dense heterochromatic structure next to the nuclear envelope out of the way of the active autosomes and the Xa. The Xi in its peripheral location and with its requirements (compared to other chromosomes) for more DNA methylation, histone deacetylation, and other epigenetic silencing biomarkers, is the last chromosome to complete replication, in the late S phase or even not until the early Gap 2 phase. This leaves the Xi vulnerable to missing check points and a possible loss of X-linked dosage compensation, in effect, potential reactivation of previously suppressed genes on the Xi since some sites of epigenetic control may fail to reestablish correctly following replication. In reality, approximately 12–20% of genes on the Xi are typically still active or potentiated for activity [79,80] and the number of genes that escape or reactivate through cell generations can increase with stress and age [81]. As a result, X-linked expression can vary from cell to cell [82]. XCI originates from the X inactivation center (XIC) at Xq13 on the long arm (Xq) (Figure 3A). The X inactivation specific transcript (XIST) gene in the XIC expresses XIST RNA, a key factor in establishing XCI. XIST RNA does not code for protein, but remains in the nucleus and binds contiguous chromatin, in effect, binds the Xi (Figure 3B). Initially, both X chromosomes colocalize their XICs and express XIST RNA but they also express the antisense TSIX gene, which leads to dsRNA degradation of the XIST and TSIX RNAs [83]. Eventually, via a stoichiometric mechanism, one X chromosome persists in expressing XIST RNA and becomes the Xi while the other X chromosome shuts off XIST expression and becomes the Xa. XIST RNA establishes XCI by binding at *LINE-1* sites along the X chromosome [84]. *LINE-1* sites on the X chromosome are at a 2x concentration (~34% of the X) relative to the overall genome average (~17%). However, on the X chromosome, the *LINE-1* concentration drops as one goes from the Xq into the Xp so that the extent of XCI is greater in the Xq relative to the Xp [85]. As the XIST RNA binds, it recruits factors involved in epigenetic silencing and chromatin structural frameworks such as: histone macroH2A; lamin-B; scaffold attachment factor A (SAF-A); transcription repressors SPEN and SMART; histone deacetylases; and other items [81,86]. In addition, the Xi’s topologically associated domains (TADs) are held as a single super-structure of heterochromatin by an architectural protein called structural-maintenance-of-chromosomes hinge domain containing 1 (SMCHD1) [87]. This provides yet another layer of epigenetic silencing of the Xi and another factor that may be disrupted in disease mechanisms. These layers, however, mean that it takes additional work to reestablish proper X inactivation as cells replicate.

The XIST RNA, *LINE-1* sites, and other factors then form a heterochromatic compartment that excludes RNA polymerase II (RNA pol II). At the core of the Xi is a dense heterochromatic mass (blue or dark color in Figure 3C–E) comprised mainly of silenced Xq genes and to a lesser extent silenced Xp genes. At the surface, euchromatic-like chromatin (yellow or light color in Figure 3E) would be comprised of those genes that remain active, are potentiated for activity, or are silent but reside next to active genes. This surface layer would consist primarily of chromatin from the short arm, Xp, since XCI in the Xp begins after much of XCI in the Xq has occurred and more genes in the Xp remain active than in the Xq. These genes at the surface would be most vulnerable to disruption, such as from a rapidly expanding nucleolus, since these primarily Xp genes are at the interface with the rest of the nuclear activity and they are the first to open in replication and last to be fully repackaged into the Xi structure following replication. 

The X chromosome has several genes that have shown significant associations with SLE and other autoimmune diseases among the approximately 1098 protein coding genes and 155 x 10^6^ base pairs of the X [85]. Table 2 lists some of these autoimmune related genes and fragile sites. The “X chromosome-nucleolus nexus” hypothesis focuses on five items in the short arm of the X chromosome: 1) A high concentration of *Alu* elements in the pseudo-autosomal region 1 (PAR1); 2) fragile site FRAXB in Xp22; 3) a functional “hot” *LINE-1* element in Xp22; 4) *spermine synthase* (*SMS*) at Xp22.1, an enzyme involved in polyamine synthesis; and 5) *spermidine/spermine N1 acetyltransferase* (*SAT1*) also at Xp22.1, an enzyme involved in polyamine recycling. These five items are located from Xp22 to the telomere of the X short arm and wind up at the euchromatic surface of the Xi. There are 29 protein coding genes in PAR1, all of which escape inactivation. Some of the genes in Xp22, which would typically be near the surface of the Xi are epigenetically silenced, such as *SMS* and *SAT1*, but they are vulnerable to reactivation since they are located between active genes on the Xi [88]. With regards to the *Alu* elements, *Alu* elements are enriched in PAR1 at 28.8% (versus 10.8% overall in the human genome), which figures to be greater than 2500 *Alu* elements in PAR1 [85]. *Alu* elements are normally kept silent by positioned nucleosomes but, if the nucleosomes are shifted or displaced, it can open an internal RNA pol III transcription site, (in effect, no 5′ promoter is needed) in the *Alu* elements and result in a rapid increase in *Alu* RNA transcripts. In addition, *Alu* elements can form cruciforms, which can interfere with repackaging of the DNA. This could be particularly problematic when exposed to high polyamine levels in nucleoli.

## 3. The “X Chromosome-Nucleolus Nexus” Hypothesis in Action

### 3.1. Disruption of the Inactive X Chromosome

**Figure 4:**
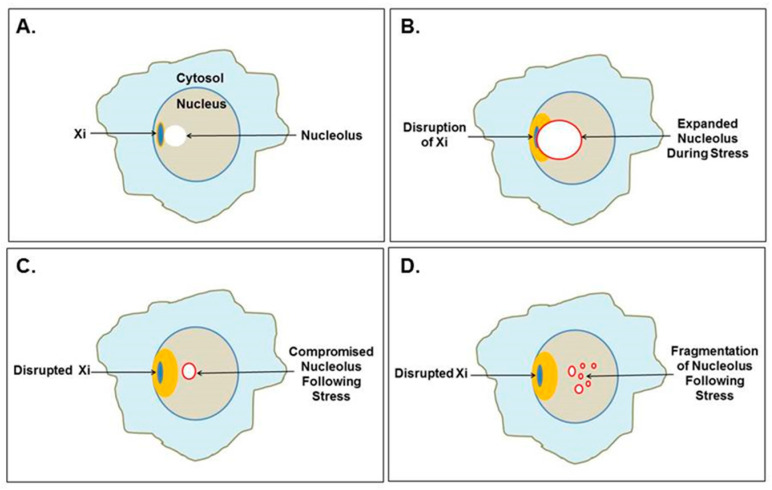
Mutual disruption of the Xi and nucleolus. (**A**) The Xi, which localizes to the nuclear envelope, is often found near a nucleolus, even as part of the nucleolar shell. (**B**) Cellular stress could cause extraordinary nucleolar expansion, facilitated by increased polyamine synthesis that disrupts the neighboring Xi. (**C**) Previously suppressed Xi genes could be opened for expression, which could include *SAT1* that alters polyamine levels affecting nucleolar functions and future nucleolar stress response. (**D**) Nucleoli could even fragment as the nucleolar shell is disrupted by RNA pol III Alu transcripts as demonstrated by Caudron-Herger et al. [25].

The Xi localizes to the nuclear envelope and is in close association with a nucleolus in one-third of cells throughout the cell cycle (except mitosis when nucleoli disappear) and it is close to a nucleolus in 90% of cells in the S phase [20]. Therefore, the Xi is frequently sandwiched between the nuclear envelope and a nucleolus (Figure 4A). This puts the epigenetically silenced Xi next to one of the most active components of the cell, the nucleolus. A recent report provides more detail to this Xi-nucleolus association finding that the Xi moves closer to the nucleolus during the G0/G1 transition [97]. In this proximal position, the nucleolus could assist in maintaining XCI, use the Xi as part of the nucleolar heterochromatic shell, facilitate replication of the Xi and, later, assemble the Xi’s centromeres preceding mitosis. However, nucleolar expansion, especially during cellular stress, such as activation of EBV or some other latent virus that stimulates increased polyamine synthesis via *c-MYC* induction, could lead to conflict between the Xi and the nucleolus (Figure 4B). The nucleolus would be attempting to restructure its heterochromatic shell, including the Xi, to enlarge the nucleolus’s volume. Particularly vulnerable to disruption would be genes in the Xp22 through PAR1 chromatin at the surface of the Xi, as we discussed previously. This could permanently disrupt the epigenetic silencing in the Xi (Figure 4C), for example, opening *SMS* and/or *SAT1* at Xp22.1, which could lead to alteration of polyamine and SAM levels. The “hot” *LINE-1* in Xp22 and other functional reverse transcriptases (such as human endogenous retroviruses many of which are located on the X chromosome) could also be opened to expression [98,99]. Since the “hot” *LINE-1* codes for a fully functional reverse transcriptase, there could be subsequent reverse transcription generating hypomethylated DNA that could provoke the immune system. Dewannieux et al. demonstrated that the *LINE-1* reverse transcription would preferentially reverse transcribe *LINE-1* RNA and Alu RNA compared to the RNA pol II transcribed RNAs [100]. The reverse transcribed *Alu* DNA (generated in the cytoplasm) would require extensive methylation since Alu elements are very rich in CpG sites but most DNA methyl transferases are localized to the nucleus. Hypomethylated *Alu* DNA could be interpreted by DNA sensing pathways as exogenous material. Disruption of the Xi could displace the positioned nucleosomes that normally suppress expression of *Alu* elements, particularly the 2500+ *Alu* elements in the PAR1. Exposure of these open *Alu* elements to high levels of polyamines in the nucleolus could allow stabilization of Z-DNA or *Alu* cruciforms that hamper reformation of nucleosomes. As polyamine levels drop and the DNA returns to B-DNA, RNA pol III (which is in abundance near the nucleolus) could compete with histones for binding and gain access to the intragenic promoters in the *Alu* elements. Opening of PAR1 could then provide an abundance of RNA pol III transcribed *Alu* RNA transcripts since RNA pol III is very processive (i.e., transcribes and reinitiates rapidly) and does not require ATP. These *Alu* transcripts could be reverse transcribed by the *LINE-1* reverse transcriptase. Li and Steinman reported that as much as 55% of free DNA in the SLE patient sera was *Alu* DNA and they suggested that the *Alu* DNA could originate from reverse transcription [101].

In addition to possible reverse transcription activity and alterations in polyamine levels that interferes with RNA folding and involves wasteful use of SAM in synthesis (*SMS*) and recycling (*SAT1*) of polyamines, there could also be, opening of fragile sites on the Xi, such as FRAXB in Xp22, which could allow reactivation of hidden viruses. Opening a fragile site can lead to DNA breaks and chromatin fragmentation. Additionally, the disruption of chromatin and individual nucleosomes, particularly in the highly condensed Xi, can release negative supercoiling stress that can flux through the DNA and form alternate DNA conformations of Z-DNA and cruciforms, which can be stabilized by polyamines, especially when exposed to increased polyamine levels in the nucleolus. Stabilized Z-DNA, cruciforms, and/or DNA breaks could hamper reestablishment of XCI and even result in chromatin fragmentation.

### 3.2. Disruption of the Nucleolus

Disruption of heterochromatin surrounding the nucleolus, including disruption of the Xi, can lead to subsequent disruption of the nucleolus. Caudron-Herger et al. reported that the nucleolar heterochromatic shell is maintained by interaction with the chromatin by a complex of nucleolin bound with RNA pol II transcripts with intronic Alu elements [25]. When they added RNA pol III transcribed *Alu* elements, even fragments as short as 20 bases, they observed compromised nucleoli with disruption of nucleolar functions and, in some cases, fragmentation of the nucleolus (Figure 4C,D). They suggested that the RNA pol III transcripts were hybridizing with the RNA pol II intronic *Alu* elements leading to RNA degradation. One could also imagine competition between the RNA pol III transcripts in abundance and the RNA pol II intronic *Alu* elements for the limited amount of nucleolin. This abundance of RNA pol III transcribed *Alu* transcripts could occur in vivo when more than 2500 *Alu* elements residing in the Xi’s PAR1 are opened to transcription following Xi disruption by the nucleolus. These *Alu* transcripts could flood the nucleolus. We should note that there are reports of an *Alu* stress response, which could involve many of the over one million *Alu* elements throughout the genome [102].

With abnormal nucleolar functioning, the folding and assembly of proteins and RNA transcripts into proper RNPs would be affected. Fragmentation of the nucleolus could leave RNA and proteins misfolded or result in an inadequate distribution of proteins and RNAs such that in some fragments the RNP assemblies are incomplete. For example, the ribosomal RNAs may be in one nucleolar fragment but the needed ribosomal proteins are in other fragments, so the assembly of ribosomes is inefficient. The improper assembly of RNPs could leave these RNPs, or the individual proteins and RNAs, in abnormal conformations that expose autoantigenic sites. These could be cryptic epitopes that have not appeared in sufficient quantities previously for the immune system to develop tolerance [103]. The immune system will initiate an autoimmune reaction when these abnormal assemblies and epitopes are exposed. Then, through epitope spreading, the immune system may lose its tolerance for the normal protein, RNA, or RNP. In this process, the reaction is initially against the abnormal epitopes. Since antibody targeting can include neighboring epitopes or overlapping of epitopes, the targeting can shift from only the abnormal epitopes, to combinations of normal and abnormal epitopes, and then to the much more abundant normal epitopes. The events that generate these abnormalities may have passed but the loss of tolerance for the normal epitopes then becomes the observable effect. As an example of how autoantibodies can vary from specific to less specific targeting, autoantibodies targeting Z-DNA appear in SLE and RA. When spermine is present, the autoantibodies show greater binding to Z-DNA [71]. This suggests that spermine may have been involved in the initial provocation of the immune system by Z-DNA but the reaction has spread to target DNA sequences in general that have the potential to form Z-DNA.

Another adverse impact on nucleolar functioning is altered polyamine levels and types. Xi disruption could open the *SAT1* gene at Xp22 on the Xi. Now there are two active copies of *SAT1* and they can be super induced. Acetylation of polyamines will increasingly affect the balance of polyamines and acetylated polyamines in the nucleolus. This could interfere with RNA folding, RNP assembly, centromere assembly, and interfere with future stress responses.

There are other problems that could arise from the Xi and/or nucleolar disruption. For example, an overabundance of *Alu* RNA transcripts in the nucleolus could compete with the 7SL RNA of the signal recognition particle (SRP), which is assembled in the nucleolus [104]. The 7SL RNA has an *Alu* domain, which localizes the SRP to the ribosome, and a signal domain, which recognizes the extracellular protein signal in an emerging nascent polypeptide (typically the first five amino acid residues) and halts translation until the ribosome, nascent polypeptide, and SRP can relocate to an SRP receptor in the endoplasmic reticulum (ER). Then, translation can continue with the polypeptide passing into the ER lumen and folding into a conformationally correct protein that will be released extracellularly. During SRP assembly, SRP9 and SPR14 proteins bind the 7SL RNAs *Alu* domain typically with SRP9/14 at 20x the amount of 7SL RNAs. However, the RNA pol III *Alu* transcripts could compete for the SRP9/14 to the extent that some 7SL RNAs do not have functional *Alu* domains that would stop translation when the amino acid signal emerges. In this situation, translation would continue with the emerging polypeptide being exposed to cytoplasmic enzymes it would not normally encounter, such as transglutaminases. This could generate abnormal proteins such as acrolein-conjugated proteins seen in SjS [72]. The incomplete SRP could still translocate the polypeptide to the ER lumen but with improper modifications since translation was not paused. On the other hand, the RNA pol III *Alu* transcripts with SPR9/14 could localize to ribosomes where they would be readily available for *LINE-1* reverse transcription, as described by Dewannieux et al. [100].

### 3.3. New Developments in the “X Chromosome-Nucleolus Nexus” Hypothesis

Since the original publication of the “X chromosome-nucleolus nexus” hypothesis in 2015, the hypothesis has continued to expand as new information is incorporated [16]. The work of Caudron-Herger et al., in which they described disruption of the nucleolus by *Alu* RNA, provided a major advancement for the hypothesis since it could explain how transient components of the nucleolus could arise as autoantigens [25]. This was incorporated into the hypothesis in 2017 [17]. Here, we will touch on some more recent concepts that can further refine the hypothesis.

One question that was not addressed previously is why a nucleolar stress response could be abnormally intense to the extent that it disrupts the Xi and other chromatin. It is possible that an initial nucleolar stress response occurs at a particularly inappropriate time in the cell cycle, such as the late S phase when replication is still in progress. This could disturb the chromatin and allow a latent virus to become active, which amplifies the initial nucleolar stress response with additional c-Myc induced polyamine synthesis. Going forward, altered polyamine levels and expression of *Alu* RNA pol III transcripts may damage the nucleolus and affect normal nucleolar functions and future stress response. Keep in mind, however, that a cell can have more than one nucleolus. Therefore, the stress response might not adversely affect all nucleoli in the cell so that there is a chance for the cell to survive. We can also think of other factors that play into a stepwise progression of problems. Of the sites mentioned in Xp22 and PAR1, opening of each of these could be a step: Opening of *Alu* elements in PAR1; opening of *SMS* in Xp22; opening of *SAT1*; opening of the “hot” *LINE-1*; opening of FRAXB with possible latent viruses; and even opening of Xq genes of the Xi. Other Xi fragile sites (FRAXA, FRAXC, FRAXD) could contain latent viruses since common fragile sites (about 50 exist in the genome) are often sites of viral integration [105] and fragile sites exhibit genomic instability [106]. As an example, FRAXB in Xp22 is a site in which HPV16 integrates and such integrations are not restricted to any one point of the cell cycle [107].

Some new factors have been reported that could have a role in the hypothesis. The newly described SETDB1 protein assists in compaction of Xi. [108]. This could be an important protein in reestablishing the Xi heterochromatin after replication and abnormalities in the expression or function of SETDB1 could be involved in the loss of X-linked dosage compensation. We may soon find other proteins and suppressing RNA transcripts that are important in controlling the Xi’s topologically associated domains and these may pose additional epigenetic vulnerabilities.

Another item of current interest in both autoimmune and cancer research that can now be incorporated into the hypothesis is the cGAS/STING pathway [109,110]. When there is an increase in dsDNA in the cytosol, such as the presence of viral DNA (PAMPs; pathogen associated molecular patterns) or abnormal self-DNA (DAMPs; danger/damage associated molecular patterns), the 2′3′ cyclic GMP-AMP synthase (cGAS) binds the DNA and generates 2′3′-cyclic GMP-AMP (cGAMP). The cGAMP then binds the stimulator of interferon genes (STING) protein, which is an ER membrane bound protein with cytosolic exposure. STING then moves from the ER to the Golgi where STING is palmitoylated. TANK-binding kinase 1 (TBK1) then phosphorylates STING followed by STING facilitated phosphorylation of interferon regulatory factor 3 (IRF3) which dimerizes, moves to the nucleus, and induces expression of interferon genes to alert the immune system [111]. There is current interest in the involvement of mutations in the DNA 3′-5′ exonuclease gene, TREX1, in autoimmune diseases [112]. The TREX1 protein degrades cytoplasmic DNA thereby avoiding activation of the cGAS/STING pathway. The mutations prevent proper TREX1 DNA degradation so that a buildup of cytoplasmic DNA can trigger cGAS. TREX1 knockout mouse models should be very informative on both the genetics and epigenetics involved in controlling the cGAS/STING pathway.

cGAS was originally thought to be a cytosolic protein serving primarily as a PAMP sensor (i.e., sensing foreign DNA) but more recently it has been reported that the majority of cGAS (potentially 95%) is nuclear and appears to be tightly tethered to heterochromatin [113,114]. Earlier studies had not used sufficiently high salt concentrations to elute cGAS from chromatin, so cGAS was not believed to be present in the nucleus in any significant amount. However, now that it is known to be nuclear, we need to consider its function as a DAMP sensor that scrutinizes chromatin for self-DNA abnormalities. This opens many interesting possibilities for cGAS involvement in autoimmune diseases that can be incorporated into the “X chromosome-nucleolus nexus” hypothesis.

Exposure of protein-free dsDNA in the nucleus is a rare event since the DNA is buried in a swarm of proteins. Most of the DNA is bound in nucleosomes, which occur on average every 200 bp. In the nucleosome, 145 bp are wrapped in 1.5 left hand turns around an octameric histone core with additional coverage of the DNA by the tails of histones H2A and H3. The linker DNA between nucleosome cores is comprised of approximately 55 bp and is saturated with proteins, such as histone H1, transcription factors, DNA modifying enzymes, histone modifying enzymes, polymerases, topoisomerases, scaffold attachment factors, and many other proteins, counter ions (e.g., polyamines), and structural RNAs. In addition, nucleosomes are stacked into higher order structures that further restrict access to the dsDNA. The inactive cGAS proteins associated with the chromatin are primarily localized to centromeres, *LINE-1* sequences, and sites of chromatin entanglement such as incomplete separation of chromatin between two daughter cells [115]. Even when an RNA polymerase is transcribing a gene, the histones maintain contact with local dsDNA shifting from DNA in front of the polymerase to DNA behind the polymerase. Likewise, when DNA polymerases are replicating the DNA, there is always an abundance of new histones available to form nucleosomes with the new DNA. Due to the importance of having sufficient histones available and to produce histones unhindered, histone genes do not have introns and the transcribed RNA does not have a poly-A tail.

**Figure 5:**
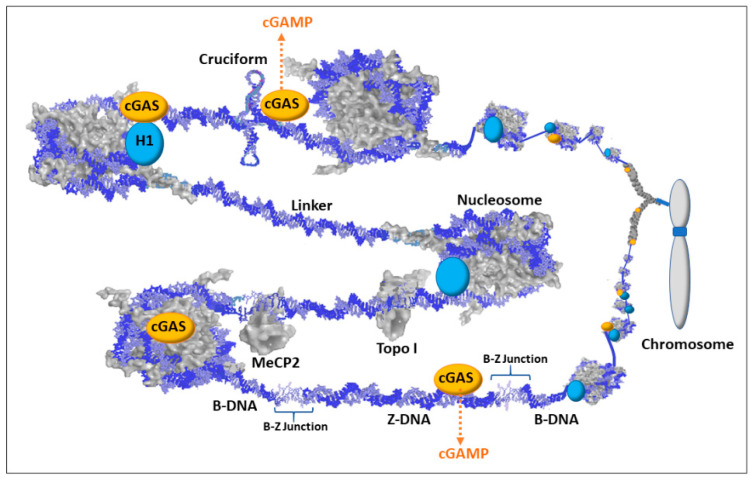
Schematic of cyclic guanosine monophosphate-adenosine monophosphate synthase (cGAS) associations with chromatin. Most dsDNA is associated with proteins such as histones (including H1), methyl capping protein 2 (MeCP2), topoisomerases (e.g., Topo I), transcription factors, scaffold attachment factors, polymerases, and centromere components. There are also structural RNAs, such as the X-inactivation specific transcript (XIST RNA) that is involved in X chromosome inactivation. Therefore, with the multiple layers of proteins and structural RNAs in chromatin, the exposure of dsDNA would be an infrequent occurrence and could signify chromatin disruption and possible underlying DNA damage. Nuclear 2′,3′-cyclic guanosine monophosphate-adenosine monophosphate synthase (cGAS) is localized to chromatin with salt-resistant interactions. This maintains cGAS in a position to bind exposed dsDNA when it occurs. The cGAS/dsDNA complex then generates cyclic guanine monophosphate adenosine monophosphate (cGAMP) that then binds the stimulator of interferon genes (STING) triggering an immune response.

Although exposure of protein-free dsDNA is a rare event, transient conformations, such as Z-DNA and cruciforms, could disrupt the chromatin and provide both stretches of exposed dsDNA and exposed single strand DNA (ssDNA). DNA binding agents and DNA intercalating agents, such as the lupus-inducing drugs procainamide and hydralazine, can also distort DNA that exposes ssDNA and dsDNA [19]. These alternate states of DNA can warn of underlying problems that could hamper replication, transcription, DNA repair, and/or proper chromatin formation, and it could indicate improper gene expression (e.g., activation of latent viral genes). Figure 5 depicts some of the scenarios involving alternate DNA conformations but keep in mind that there would be layers (not shown) of proteins and structural RNAs in the milieu jostling for contact with the DNA or their specific protein–protein interactions. In the case of Z-DNA, the dsDNA in a left-handed coil could be left exposed since it could alter the recognition sites for sequence specific binding proteins and the Z-DNA, being too stiff to bend around a histone octameric core, could hamper nucleosome reformation. In addition, the B-Z junctions between the Z-DNA and adjacent B-DNA can be from 1 to 12 bp of strand separation that is vulnerable to single and double strand breakage and mutations. Cruciforms would have exposed dsDNA extending out at right angles from the main stretch of DNA and the cruciforms would have exposed ssDNA at the looping end of its extended arms and at the junction where the cruciform joins the main stretch of DNA. The cruciforms may be difficult to methylate following replication, especially in *Alu* elements which have a very high CpG content and readily form cruciforms when open. Polyamines, especially from the high levels in the nucleolus, could bind these transient conformations and aid in stabilization and persistence. Since cGAS is localized to chromatin, the highly positively charged N terminal of cGAS could bind the Z-DNA or cruciform or stretches of DNA altered by intercalated drugs (e.g., hydralazine). The cGAS/Z-DNA, cGAS/drug intercalated DNA, and/or cGAS/cruciform complexes could potentially be stable enough to prevent or hamper attempts at resolution, such as nucleosome reformation, until the problem with the underlying DNA is resolved. Meanwhile, the cGAS would be activated (by dimerizing with another potentiated cGAS) and generate cGAMP as a DAMP signal that triggers STING and eventually the activation of interferon genes to alert the immune system. With regards to the “X chromosome-nucleolus nexus” hypothesis, Xi chromatin, especially from Xp22 through PAR1 (i.e., the end of the Xi’s short arm), would be particularly vulnerable to dsDNA exposure and alternate conformations.

First, the X chromosome is 34% *LINE-1* as opposed to the genome average of 17% [85]. This means there could be more cGAS associated with the X chromosome since cGAS associates with chromatin at *LINE-1* sites.

Second, the close proximity of the Xi to the nucleolus could lead to extensive disruption during nucleolar stress, especially if it coincides with DNA replication and reestablishment of Xi heterochromatin. This disruption could expose the Xi to high levels of polyamines in the nucleolus, which could stabilize alternate DNA conformations [116]. Moreover, the disruption would release extensive amounts of negative supercoiling stress stored in nucleosomes in the Xi heterochromatin. This released stress could flux through the chromatin and flip DNA into Z-DNA, cruciforms, and strand separation but resolution by nucleosome reformation could be difficult due to the competition between nucleosomes and stabilized Z-DNA and cruciforms for negative supercoiling stress.

Third, the very high concentration of *Alu* elements in the PAR1 region (28.8% vs. the genome average of 11%) [85] would provide many sites at which cruciforms could form if nucleosomes are displaced, and the cruciforms could be relatively difficult to methylate, leaving hypomethylated CpG rich *Alu* DNA. This could be a source of hypomethylated CpG rich dsDNA that binds TLR9 [117]. Additionally, this DNA (as well as reverse transcribed *Alu* DNA) could be a source of the large amounts (up to 55% versus genome average of 11%) of *Alu* DNA in the free DNA in sera of lupus patients reported by Li and Steinman [101].

Fourth, disruption of chromatin could expose fragile sites, such as FRAXB (fragile site B) in Xp22 in the Xi. Double stranded breaks could create chromatin fragments separated from their chromosomes and those fragments could be improperly distributed in mitosis. Nucleolar stress could be especially damaging in fragile sites. The chromatin fragments could exit the nucleus during mitosis or in micronuclei, similar to the process of chromothripsis in cancer by which chromatin can be lost or rearranged via micronuclei [118]. Chromatin fragments, such as FRAXB to the Xp terminus, with cGAS/Z-DNA and/or cGAS/cruciforms could be released in micronuclei. The Z-DNA and/or cruciform could be stabilized by high levels of polyamines and cGAS and persist when the DNA binds TLR9 in micronuclei and eventual extracellular exposure. In the autoimmune disease scenario, this can present the problematic DNA in a state similar to the abnormal context (sequence, conformation, bound proteins, bound cations) in which it was first encountered. This would allow more accurate immune scrutiny and response to the initial DAMP provocation.

## 4. Conclusions

The “X chromosome-nucleolus nexus” hypothesis is a very complicated hypothesis that continues to evolve as new pertinent research reports are incorporated. In fact, this hypothesis is actually a collection of hypotheses about the involvement of polyamines, *Alu* elements, the Xi, nucleoli, cGAS/STING, etc. The hypothesis is discussed primarily with regards to SLE as a prototypic autoimmune disease, but it can have some relevance to other autoimmune diseases. Since the “X chromosome-nucleolus nexus” hypothesis is complicated and continues to evolve, one should read earlier versions of the hypothesis and related topics [15,16,17,62,104,119], which have more detailed explanations of some of the issues discussed here.

One of the benefits of continued development of the hypothesis is that it gives us ideas of new therapeutic targets and how they might be involved in the disease. These therapeutic targets can provide earlier interdiction points in the diseases as opposed to current approaches that aim for immune suppression after the disease onset. For example, there is a keen interest now in identifying antagonists for the cGAS/STING pathway. Additionally, the hypothesis provides ideas of how endogenous DNA and/or RNA out of their proper context (e.g., altered conformations) could be interpreted as foreign material by the cGAS/STING pathway. Some other potential therapeutic targets for treating autoimmune diseases, such as SLE, are the polyamine synthesis (*SMS*, *ODC*, *AMD1*) and recycling (*SAT1*) enzymes, transglutaminases, peptidyl arginine deiminases (related to NETosis [118]), and EBV genes.

Another benefit of the hypothesis is that it attempts to present a comprehensive explanation of SLE. Many research projects are focused on finding one or a few genes of significance in SLE. Results from those projects provide only limited insights within the scope of those genes. This limits the thinking primarily to genetic concepts (e.g., gene mutations, deletions, insertions, duplications). A hypothesis, such as the “X chromosome-nucleolus nexus” hypothesis, that includes epigenetic perspectives, and not just the genetics of epigenetic biomarkers, can point to links between otherwise distant events. For example, the report of high levels of *Alu* DNA in the free DNA in SLE sera [101] suggests the possible involvement of reverse transcription in SLE but it also points to the RNA pol III transcription of sequences that are not protein-coding genes, such as *Alu* elements. Additionally, it brings up the involvement of *Alu* elements in nucleolar integrity and disruption and the concept of alternate DNA conformations such as Z-DNA and *Alu* cruciforms. Then, this gets into the dynamics of epigenetics and DNA supercoiling stress, topics that do not get discussed enough even within the field of epigenetics.

The hypothesis provides some explanations relative to SLE for disease features such as female predominance (Xi involvement), primarily adult onset (accumulation of epigenetic changes from multiple stress events), occurrence of antinuclear autoantibodies (chromatin disruption and DNA sensors, such as cGAS), and occurrence of some cytosolic autoantibodies (nucleolar disruption that leads to abnormal RNPs, such as ribosomal subunits, that are supposed to function in the cytosol). These disruptive events can also apply to infrequent autoimmune reactions arising in cancers and cancer therapy. The hypothesis can also provide some explanation for polyautoimmunity, such as SLE patients with SjS. Both of these diseases appear to be impacted by polyamines (e.g., spermine involvement in autoantigenic DNA in SLE and acrolein conjugated proteins in SjS).

The hypothesis requires understanding of a broad range of fields of study, but most readers would have only limited knowledge with regards to one or more of the areas, such as DNA supercoiling, SLE, chromatin structure, X chromosome inactivation, nucleolar functions, etc. Presenting the hypothesis as it evolves is an ongoing attempt to bring these together into an increasingly comprehensive explanation. Of course, as a hypothesis or collection of hypotheses, some aspects may be disproven or need refinement. As it is, there are highly capable research teams in each of the fields continually providing reports on basic cell biology, new findings regarding autoimmune diseases, and new methods to gather data on intracellular events during stress. Their findings will help with further refinements of the hypothesis. The hope is that readers will want to discuss the hypothesis to assist in refining it and perhaps perform experiments within their area of expertise that can support or disprove parts of the hypothesis. Particularly interesting will be experiments on long distance chromatin interactions, such as in the nucleolar heterochromatic shell under stress using 3C (chromatin conformation capture analysis) and 4C (circularized chromatin conformation capture analysis). This could demonstrate the extent of changes that could correlate with other activities, such as increased polyamine synthesis and cGAS/STING activation.

## Figures and Tables

**Table 1:** Nucleolar related autoantigens in systemic lupus erythematosus (SLE).

Autoantigen	Function/Complex	Occurrence (% in SLE)	Reference
Nucleolin	Nucleolar structural integrity	>50	[26]
U1RNP	Spliceosome component	40	[26]
U1RNA	Spliceosome component	<5	[26]
Sm epitopes	Spliceosome proteins	25	[26]
SSA/Ro	RNA pol III chaperone	40–50	[26]
SSB/La	RNA pol III chaperone and termination	15	[26]
Ribosomal P proteins	Phospho proteins, bind 28S RNA	12–16	[27]
Ku	dsDNA break repair	20–40	[26]
Cardiolipin	Similar epitopes to nucleophosmin	20–40	[26]
Centromere components	CENP-B and others	~6	[26]
Lamins	Complexed with nucleolin	unknown	[28]

**Table 2:** X-linked genes and fragile sites associated with autoimmune diseases.

Gene/Site	Name	Location	Potential Issue	Reference
*Alu* elements	Short Interspersed Elements	Enriched in PAR1	Disruption	[15]
FRAXB	Fragile Site B	Xp22	Latent viruses, DNA damage	[89]
(hot) *LINE-1*	Long Interspersed Elements	Xp22	Reverse transcription	[15]
*SMS*	*Spermine Synthase*	Xp22	Polyamine dysregulation	[15]
*SAT1*	*Spermidine/Spermine N1 Acetyltransferase*	Xp22	Polyamine dysregulation	[15]
*TLR7*	*Toll-like Receptor 7*	Xp22	Overexpression	[90]
*FOXP3*	*Forkhead Box P3*	Xp11	T-cell dysregulation	[91]
*CXCR3*	*C-X-C motif chemokine receptor 3*	Xq13	Overexpression	[92]
FRAXC	Fragile Site C	Xq22	Latent viruses, DNA damage	[89]
*CD40L*	*Cluster of differentiation 40 ligand*	Xq24	Overexpression	[90]
*HERV-w*	*Human endogenous retrovirus w*	Xq22	Dysregulation	[93,94]
FRAXD	Fragile Site D	Xq27	Latent viruses, DNA damage	[89]
*MeCP2*	*Methyl-CpG-binding protein 2*	Xq28	DNA methylation dysregulation	[95]
*IRAK1*	*Interleukin-1 receptor associated kinase-1*	Xq28	Checkpoint dysregulation	[96]
FRAXA	Fragile Site A	Xq28	Latent viruses, DNA damage	[89]
